# The Treatment of Fracture

**Published:** 1897-07-17

**Authors:** 


### The Treatment of Fracture.

Recent writers on the subject of fractures raise the question whether the treatment of this class of accident has participated in the general advance in surgery as fully as it should have done.

Mr. Arbuthnot Lane shows in how large a proportion of cases of oblique fracture of both bones of the leg the patient suffers permanent deterioration of his earning power. Regarding him as a wage-earning mechanism, he says: "His machinery is financially depreciated by the accident, occasionally to the extent of at least 70 or 80 per cent. of its original value." This should not be, for except in the rarest cases nature is quite capable of producing firm union and of restoring to the limb its full functional capacity on the one condition that the broken fragments are replaced in their original position. What the surgeon has to do is a mere mechanical affair of fitting the fragments together and holding them in place. Yet this "setting" of a bone is a very difficult matter if perfection is to be aimed at. Moreover, it is a very painful matter, and we would hazard the conjecture that, while the unwillingness of surgeons to cause pain has not infrequently conduced to non-success, some of the success which certain bone-setters are asserted to have obtained has been due to the fact pain was part of their stock-in-trade, the means by which they impressed the bystanders, and that by sheer force, combined with complete indifference to the resulting agony, they have sometimes been able to unlock impactions, and by restoring bones to their correct positions to get good results.

We cannot but agree with the proposition strongly laid down by Mr. Pearce Gould that it is the first duty of the surgeon to obtain at once a complete and perfect reduction of the fragments. Those who have most frequently opened up compound fractures and seen the complicated condition of affairs existing within them will be the first to admit the importance of getting the fragments into position at the earliest possible moment, and to recognise that the lapse of time must always increase the difficulties in the way of so doing, and must often render them insuperable. In view, then, of the fact that the whole future of the case depends on immediate reduction being performed with complete thoroughness, and recognising on the one hand the great painfulness of the process, and on the other the great obstruction to its due performance offered by the involuntary resistance of the patient, the aid of an anæsthetic should certainly be sought in every case of difficulty. Mr. Pearce Gould urges the necessity of administering an anæsthetic as an aid both to the diagnosis and treatment of all but the simplest cases of fracture. Another thing he insists on, which, we fear, is too often overlooked, viz., that the proper use of splints is to keep bones in the position in which they have been placed, not to force them into their correct position.

A much more serious question now has to be considered. If it once be admitted that the accurate coaptation of the fragments is a matter of preponderating importance, we must go further than the mere administration of an anæsthetic; and the question of performing an operation with the object of accurately adjusting the broken bones and fixing them in position by wires or screws forces itself to the front.

Nothing could show more forcibly the complete change which has come over the point of view from which surgical problems are regarded than the fact that surgeons are now gravely recommended to open up simple fractures, making them compound merely for the sake of securing perfect adaptation of the fragments. Yet that is the point to which we have arrived; and in any case in which the fragments do not fall into satisfactory position, the question of operation has now to be duly considered.

Mr. Pearce Gould says that the cases in which it is impossible to obtain exact replacement and fixation by aid of an anæsthetic are but few; but that in those few cases, if the surgeon has the requisite means at command, and if he feels a reasonable certainty that the difficulties in the way of reduction can be overcome by operation, then it is his duty to operate.

The position taken up by Mr. Arbuthnot Lane is, however, much stronger, and, if we admit his premisses, more logical. He asserts without hesitation that in the case, for example, of simple oblique fractures of the lower extremity, thorough and complete setting of the fracture cannot be brought about by manipulation. It is not surprising, then, that he recommends operation. He points out the absurdity of a surgeon refraining from operating in a case of Pott's fracture on the ground that it is unjustifiable to convert a simple into a compound fracture, while all the time he would not hesitate to lay open the joint for the sake of wiring the patella. What has, however, to be borne in mind is that replacement of the fragments is often far from a simple matter, and that the certainty of being able to fix them in position is not such as to warrant us in advising a course which involves risk. The essence of the whole problem lies in the fact that an aseptic operation does not involve risk. The very first duty of a surgeon is, then, so to train himself and his assistants in antiseptic methods as to ensure an aseptic course to all wounds for which he is responsible. This done, the whole range of surgery is open to him; while, till this is accomplished, he cannot with safety even open an abscess.

